# [Retracted] Puerarin improves graft bone defect through microRNA-155-3p-mediated p53/TNF-α/STAT1 signaling pathway

**DOI:** 10.3892/ijmm.2024.5404

**Published:** 2024-07-22

**Authors:** Yang Zhou, Hongyu Lian, Kexin Liu, Deli Wang, Xuelian Xiu, Zhang Sun

Int J Mol Med 46: 239-251, 2020; DOI: 10.3892/ijmm.2020.4595

Following the publication of the above paper, it has been drawn to the Editor's attention by a concerned reader that the immunohistochemical assay data shown in Fig. 4B on p. 245 were strikingly similar to data appearing in different form in another article written by different authors at different research institutes that had already been published in the journal *International Journal of Biological Sciences* prior to the submission of this paper to *International Journal of Molecular Medicine*. In view of the fact that the contentious data had already apparently been published previously, the Editor of *International Journal of Molecular Medicine* has decided that this paper should be retracted from the Journal. After having been in contact with the authors, they agreed with the decision to retract the paper. The Editor apologizes to the readership for any inconvenience caused.

